# Global analysis of saliva as a source of bacterial genes for insights into human population structure and migration studies

**DOI:** 10.1186/s12862-014-0190-3

**Published:** 2014-08-22

**Authors:** Karsten Henne, Jing Li, Mark Stoneking, Olga Kessler, Hildegard Schilling, Anne Sonanini, Georg Conrads, Hans-Peter Horz

**Affiliations:** 1 Division of Oral Microbiology and Immunology, Department for Operative Dentistry, Periodontology and Preventive Dentistry, RWTH Aachen University Hospital, Pauwelsstrasse 30, Aachen, D-52057, Germany; 2 Department of Evolutionary Genetics, Max Planck Institute for Evolutionary Anthropology, Deutscher Platz 6, Leipzig, D-04103, Germany; 3 Current address: Max Planck Independent Research Group on Population Genomics, Chinese Academy of Sciences and Max Planck Society Partner Institute for Computational Biology, Shanghai Institutes for Biological Sciences, Chinese Academy of Sciences, Shanghai 200031, China; 4 Division of Virology, Institute of Medical Microbiology, RWTH Aachen University Hospital, Pauwelsstrasse 30, Aachen, D-52057, Germany

**Keywords:** Oral Microbiome, Human migration pattern, Glucosyltransferase

## Abstract

**Background:**

The genetic diversity of the human microbiome holds great potential for shedding light on the history of our ancestors. *Helicobacter pylori* is the most prominent example as its analysis allowed a fine-scale resolution of past migration patterns including some that could not be distinguished using human genetic markers. However studies of *H. pylori* require stomach biopsies, which severely limits the number of samples that can be analysed. By focussing on the house-keeping gene *gdh* (coding for the glucose-6-phosphate dehydrogenase), on the virulence gene *gtf* (coding for the glucosyltransferase) of mitis-streptococci and on the 16S-23S rRNA internal transcribed spacer (ITS) region of the *Fusobacterium nucleatum/periodonticum*-group we here tested the hypothesis that bacterial genes from human saliva have the potential for distinguishing human populations.

**Results:**

Analysis of 10 individuals from each of seven geographic regions, encompassing Africa, Asia and Europe, revealed that the genes *gdh* and ITS exhibited the highest number of polymorphic sites (59% and 79%, respectively) and most OTUs (defined at 99% identity) were unique to a given country. In contrast, the gene *gtf* had the lowest number of polymorphic sites (21%), and most OTUs were shared among countries. Most of the variation in the *gdh* and ITS genes was explained by the high clonal diversity within individuals (around 80%) followed by inter-individual variation of around 20%, leaving the geographic region as providing virtually no source of sequence variation. Conversely, for *gtf* the variation within individuals accounted for 32%, between individuals for 57% and among geographic regions for 11%. This geographic signature persisted upon extension of the analysis to four additional locations from the American continent. Pearson correlation analysis, pairwise Fst-cluster analysis as well as UniFrac analyses consistently supported a tree structure in which the European countries clustered tightly together and branched with American countries and South Africa, to the exclusion of Asian countries and the Congo.

**Conclusion:**

This study shows that saliva harbours protein-coding bacterial genes that are geographically structured, and which could potentially be used for addressing previously unresolved human migration events.

## Background

The human body and its commensal microbiota represent a highly integrated “superorganism”, which is the result of a long-term co-evolution. It is generally recognized that the structure and diversity of the human microbiome is of medical importance not only at the species level but also at the intra-species level given that bacterial strains or clonal variants thereof can differ greatly in virulence [1],[2]. While clonal types can vary among individuals they may be quite similar among family members [2]. The fact that human history is characterized by numerous migration events with the microbiome being a permanent companion suggests that the global distribution of bacterial clones is likely to be biogeographically structured. The concept of a shared history and co-migration between humans and microbes has been particularly exemplified in the case of *Helicobacter pylori*, whose genetic diversity is geographically and ethnically well structured [3],[4]. For instance, genetic variation in *H. pylori* exhibits a signature of a recent African origin followed by clonal spread across Europe and Asia, in excellent agreement with inferences from human DNA markers [3]. In addition to providing independent evidence for previously-inferred human migrations and relationships, studies of *H. pylori* have helped resolve controversies concerning particular issues. For example, a recent study of *H. pylori* found strong evidence that Polynesians trace their ancestry specifically to Taiwan [5], a view favored by some studies of the human genetic evidence [6],[7] but not others [8],[9]. Moreover, a study of *H. pylori* was able to provide insights into the history of two religious communities in India that could not be distinguished based on human DNA markers [10], presumably because of the recent time scale involved. Despite the value and the background knowledge that already exists for *H. pylori*, this organism has two major drawbacks. Studies of *H. pylori* require stomach biopsy material, which severely limits the populations that can be sampled. In addition, *H. pylori* is present only in about 50% of the human population [11], and this prevalence is constantly decreasing [12].

Thus, the identification of potentially informative and easily-accessible species for studies of human population history would be highly valuable. The claim for convenient sampling and for a high number of bacterial specimens instantly draws attention to the oral cavity and especially to saliva, which is easily collected non-invasively, without causing pain, discomfort or embarrassment to the individual. In addition, the salivary microbiome constitutes a rich source of hundreds of very diverse bacterial species reflecting the numerous oral niches, such as the tongue, tonsils, throat, hard and soft palate, buccal surfaces and gingivae [13]. Furthermore, members of the oral microbiome are stably preserved for millennia in calcified dental plaque [14],[15], which means that the establishment of suitable oral bacteria or their genes out of saliva may also support the targeted analysis of historic and prehistoric dental calculus of our ancestors. Lastly, saliva is increasingly preferred for sampling as a source of human DNA for epidemiologic and population genetic studies [16].

Whether or not the genetic diversity of oral species may allow distinction of human populations strongly depends on two requirements: first is the degree by which the bacteria are transmitted vertically (i.e. from parents to their children), and such evidence is provided by a number of studies [17]–[20]; second is the degree by which variations arising from mutation are confined to the descendants of the bacterial cell in which they occurred (such a so-called clonal structure would require that genetic exchange is sufficiently rare so that it does not affect population structure). In fact, evidence of a clonal structure and associations between oral bacterial strains and their human host populations exist for a number of species, e.g. streptococci [21], *Fusobacterium nucleatum*[22], *Aggregatibacter actinomycetemcomitans*[23], and *Haemophilus influenzae*[24].

However as with *H. pylori*, epidemiological data from the above oral species stem generally from clinical isolates grown in routine laboratories for diagnostic purposes. This means that each patient or individual is represented by a single bacterial isolate (i.e. single clone type) as usually routine microbial diagnosis does not account for multiple clones of an identified bacterial species. Given that bacterial strains are often split into numerous clones within the same individual [2], it is legitimate to ask how well a single clone of a bacterial strain represents its host. In addition it is not known (or has not been reported) whether diagnostic laboratories from different areas or countries employ different sampling and cultivation techniques, which may lead to the preferential isolation of certain clonal types over others. Such a possible cultivation bias may contribute to some extent to the genetic variations that has been observed across geographic regions. Therefore, in order to systematically elucidate whether oral bacteria have a biogeographic structure, we here analyzed bacterial genes from the saliva of 120 healthy individuals that were sampled from 12 world-wide regions covering the Northern and South American continent, Europe, Asia and Africa [25]. Based on a non-culture strategy (i.e. PCR amplification directly from salivary DNA-extracts followed by cloning and sequencing) our aim was to analyze and compare gene variation of multiple clones per individual. Instead of a combined analysis of a set of 6 to 8 house keeping genes, as is usually performed with multi-locus sequence typing (MLST) for characterizing bacterial isolates [26], we examined genetic variation within single genes, thereby accounting for the multiple clonal types that exist within individuals. For this purpose we selected three different gene types that are likely to undergo different mutation rates, and consequently may exhibit different discriminatory power among bacterial strains. With a focus on the mitis-streptococci group we used a partial stretch of the “housekeeping gene” *gdh*, encoding for glucose-6-phosphate dehydrogenase [27],[28], and of the virulence gene *gtf*, encoding the glucosyltransferase involved in dental biofilm formation [29]. In addition, we analyzed the 16S-23S rRNA internal transcribed spacer region (ITS) of *Fusobacterium nucleatum*/*periodonticum*[30],[31]. The above species were initially selected because they are ubiquitous and easily detectable in human saliva, and distinguishable down to sub-species and clonal-cluster level [2],[22]. Furthermore, these species are either pioneers of biofilm formation (i.e. mitis-streptococci), or important bridging organisms at more advanced biofilm stages (i.e. *F. nucleatum*) which means that there might exist a more or less tight evolutionary relationship between these commensals and their host. While the *gdh* gene is generally under stabilizing selection for conserving important metabolic functions [32], the ITS may evolve largely neutrally, as mutations at most nucleotides sites lead to only low functional constraints of the transcribed molecule [33]. The overall evolutionary pressure that acts upon the virulence gene *gtf* may range from stabilizing to positive selection depending on the individual site (e.g. its catalytic domain has recently been reported to be under stabilizing selection [34]). However we restricted the analysis to a rather non-conserved region [35],[36], which may also evolve largely under neutrality. In this pilot study we show that sequence data sets generated from oral bacterial genes differ in their potential to display human population relationships, with *gtf* exhibiting a geographic signature comparable to the genetic structure of human populations from different continents.

## Methods

### DNA samples, PCR amplification, cloning and sequencing

DNA-extracts from saliva samples were available from a previous study, in which sample details are described [25]. Briefly, saliva samples encompassed 10 individuals from each of 12 locations and were collected with informed consent. The criteria for selection of individuals were that individuals should not be from the same household, should not exhibit or report any oral diseases, and they should not have been away from the sampled location within the past two months [25]. The 12 geographic regions were: Argentina (AR), Bolivia (BO), California (CA), China (CH), Congo (CO), Georgia (GE), Germany (DE), Louisiana (LO), Philippines (PH), Poland (PO), South Africa (SO), and Turkey (TU), Additional file 1: Figure S1.

The three target genes (*gdh*, *gtf*, and ITS) were PCR-amplified directly from DNA-extracts with primers and thermal profiles specified in Table 1. The PCR assay for *gdh* (encoding the glucose-6-phosphate dehydrogenase gene) covered the three species, *Streptococcus oralis,* and two closely related species *S. mitis,* and *S. infantis*[2]. The PCR assay for *gtf* (encoding the glucosyltransferase gene) was specific to the *gtfR* of *S. oralis*[35], and the PCR assay for the 16S-23S rRNA internal transcribed spacer region (ITS) covered *Fusobacterium nucleatum and* the closely related *F. periodonticum*. For amplification of the ITS the forward primer (targeting a specific region of the 16S rRNA gene) was designed by the “probe design” tool of the ARB software package [37]. The reverse primer (targeting a specific region of the 23S rRNA gene) was designed manually based on a conserved region of publicly available 23S rRNA gene sequences. The sensitivity and specificity of both primers was verified separately through “test probe” using SILVA [38].

**Table 1:** Primer description

	**Primer names**	**Sequence (5' - 3')**	**Reference**	**Target gene and group**	**Thermal profile**^**1**^**(°C)**	**Amplicon size (size of analyzed fragment**^**2**^**)**
1	gdhF	ATGGACAAACCAGCNAGYTT	[2]	*gdh* gene of *S. oralis*, *S. mitis* and *S. infantis*	94°C,30s	ca. 659 bp
					55°C,30s	
						(619 bp)
	gdhR	GCTTGAGGTCCCATRCTNCC			72°C,60s	
2	MKR-F	TCCCGGTCAGCAAACTCCAGCC	[35]	*gtfR* of S. oralis	94°C,30s	ca. 374 bp
					66°C,30s	
						(330 bp)
	MKR-Rv	GCAACCTTTGGATTTGCAAC			72°C,60s	
3	FN1164F16S	CGATGAGTAGGAGGAAGG	This study	16S-23S rRNA-ITS-gene of *F. nucleatum* and *F. periodonticum*	94°C,30s	ca. 637 bp
					52°C,30s	
					72°C,60s	
						(252 bp, incl. gaps)
	FN121R23S	CATTCGGAAATCCTAGAYTCTT				

^1^All PCR-assays started with an initial denaturation at 94°C for 2 min, followed by 35 cycles of the thermal profile as given in the table, with a final elongation phase at 72°C for 15 min (s = seconds).

^2^For analysis the primer region was omitted in the case of *gdh* and *gtf*, and in the case of the ITS the flanking 16S rRNA and 23S rRNA gene regions were omitted.

PCR amplification products were visualized by electrophoresis on a 1% agarose gel supplemented with ethidium bromide. PCR products were subsequently ligated into the pCR 4-TOPO cloning vector (Invitrogen) and cloned into *Escherichia coli* One Shot chemocompetent cells (Invitrogen). Cloning and transformation reactions were performed as described by the supplier. Ten to 15 clones were picked at random from each library and bi-directionally sequenced with the plasmid primers M13.

### Data analysis

Raw sequences were quality checked manually using the program VectorNT. Sequence alignment was performed with MAFFT [39]. For the analysis of the ITS the co-amplified 16S rRNA and the 23S rRNA gene region were initially used for establishment of a proper alignment and subsequently removed for further analysis. Determination of the start and end points of the ITS region was performed as described previously [30]. ITS-sequences containing tRNAs were not used for further analysis. Sequence clustering into unique sequence types and definition of operational taxonomic units (OTUs) based on a 99%-identity level was performed with mothur [40]). Coverage of OTUs per geographic location was assessed based on the nonparametric estimator of total richness using Chao1 [41]. Pair-wise Fst values as well as the analysis of molecular variance (AMOVA) were calculated in Arlequin (version 3.0) under default settings [42]. Resulting distance matrices were then exported into MEGA 5 [43], and used for Cluster analysis (UPGMA or neighbor-joining). Based on the OTU-distribution the Pearson distance correlations were calculated and resulting distance matrices used for cluster analysis with MEGA 5. Sequence information was also used for phylogenetic tree reconstruction (UPGMA or neighbor-joining) based on MEGA 5 and interpretation of phylogenetic trees (Cluster analysis and principal coordinate analysis) performed with FastUniFrac [44]. Haplotype network analysis was performed with Network, version 4.612 [45]. Correspondence analysis was performed with the “ca”-package in R in analogy to a recent study in which the genetic diversity of the human polyomavirus JC was used for reconstructing human migrations [46]. Sequences determined in this study have been deposited to Genbank and are available under the acc.-no. KM074060 - KM075227 (*gtf*), KM075228 - KM075859 (*gdh*), and KM075860 - KM076596 (ITS).

This study was approved by the Ethics Commission of the University of Aachen, Medical Faculty, reference number EK167/09.

## Results

Saliva samples from ten individuals each from seven countries (i.e. CH, CO, DE, GE PH, PO, TU) were initially selected for analysis. For all three target genes PCR amplicons of correct fragment size were obtained in the majority of cases (i.e. 65/70 for *gdh*, 64/70 for *gtf*, and 68/70 for ITS). Target specificity was verified through comparison with the GenBank database and based on tree reconstructions using publicly available reference sequences (data not shown). Generally 9 to 10 individuals tested positive in each country, except for Georgia which showed the lowest number of positive cases (e.g. only 6/10 for *gtf*, Table 2). Approximately 700 sequences were analyzed from generated clone libraries for each of the three genes. An overview of the number of individuals, sequences, OTUs and coverage of diversity based on the Chao 1 Richness Estimator is provided in Table 2. The highest number of unique sequence types and OTUs defined at the 99%-identity level were obtained for the *gdh*, followed by ITS and *gtf*. At the OTU level, coverage of genetic diversity per country ranged from 28% to 66% (*gdh*), from 65% to 100% (*gtf*), and from 43% to 63% (ITS), respectively.

**Table 2:** Statistics for the analyzed individuals and sequences

**Gene**	***gdh***					
**Group**^**1**^	**# Individuals**	**# Sequences**	**# Unique sequences**	**# OTUs (99%-level)**	**CHAO1 Estimated richness**	**Percentage coverage**
CH	9	113	76	57	103	55
CO	9	92	73	57	116	49
DE	9	89	71	52	131	40
GE	8	52	46	39	101	39
PH	10	104	92	78	280	28
PO	10	113	83	59	118	50
TU	10	69	52	41	62	66
Total	65	632	484	361	797	45
**Gene**	***gtf***					
**Group**^**1**^	**# Individuals**	**# Sequences**	**# Unique sequences**	**# OTUs (99%-level)**	**CHAO1 Estimated richness**	**Percentage coverage**
CH	9	113	29	10	10	100
CO	9	99	28	10	10	100
DE	10	106	40	6	6	100
GE	6	71	24	11	17	65
PH	10	111	37	10	12	83
PO	10	110	39	11	14	79
TU	10	110	43	11	17	65
Total	64	720	213	31	38	82
**Gene**	**ITS**					
**Group**^**1**^	**# Individuals**	**# Sequences**	**# Unique sequences**	**# OTUs (99%-level)**	**CHAO1 Estimated richness**	**Percentage coverage**
CH	10	114	73	54	120	45
CO	9	97	67	57	143	40
DE	10	94	39	28	63	44
GE	9	98	69	54	124	43
PH	10	117	80	61	143	43
PO	10	108	59	47	82	57
TU	10	108	55	39	62	63
Total	68	736	321	203	412	49

^1^CH = China, CO = Congo, DE = Germany, GE = Georgia, PH = Philippines, PO = Poland, TU = Turkey.

The number of polymorphic sites for each gene was 363, 68, and 200 for *gdh*, *gtf*, and ITS, respectively. Compared to the lengths of the analyzed gene region, the proportion of polymorphic sites thus was 59% for *gdh*, 21% for *gtf*, and 79% for the ITS.

**Figure 1:**
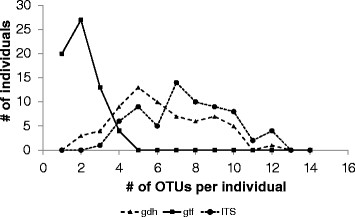
**Comparison of the number of different OTUs per individual among the genes*gdh*,*gtf*, and ITS.** Results are depicted from the individuals of the seven countries CH, CO, DE, GE PH, PO, TU. CH = China, CO = Congo, DE = Germany, GE = Georgia, PH = Philippines, PO = Poland, TU = Turkey.

Generally multiple gene variants were found in each individual, with the majority of people exhibiting five to ten different OTUs in the case of *gdh* and ITS (Figure 1). This was different for the *gtf* as most individuals were characterized by only one or two different OTUs. A maximum of four OTUs was found in just four individuals (Figure 1).

**Figure 2:**
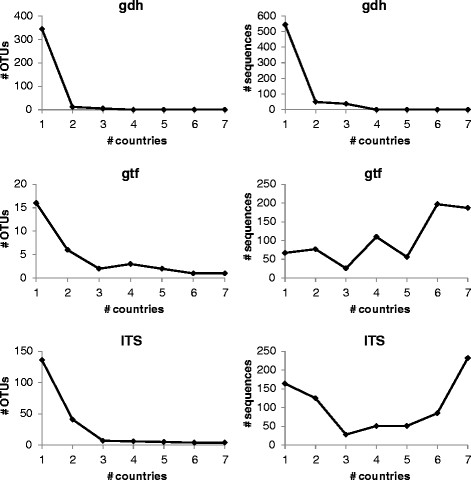
**Distribution of OTUs (left panel) and corresponding number of sequences (right panel) across countries.** Results are depicted from the individuals of the seven countries CH, CO, DE, GE PH, PO, TU. CH = China, CO = Congo, DE = Germany, GE = Georgia, PH = Philippines, PO = Poland, TU = Turkey.

Classification of the OTUs according to their occurrence in a single or in multiple countries (i.e. co-occurrence in any two countries, any three countries and so on) resulted in very different curve progressions for the three genes. The left panel of Figure 2 displays the type of OTU (i.e. prevalence across countries), while the right panel indicates the number of sequences for each OTU-type. The majority of OTUs were found to be restricted to one particular country in the case of *gdh* and ITS (95% and 67%, respectively). For *gdh* those country-specific OTUs were represented by 544 sequences (86% of all sequences), and for ITS by 164 sequences (22% of all sequences). The remaining 5% of *gdh*-OTUs were distributed over a maximum of three countries. The curve of the ITS-OTUs was characterized by a less steep gradient, with 41 OTUs (20%) shared between any two countries, and 26 OTUs (13%) shared among multiple countries. Four ITS-OTUs were distributed over all seven countries, and were represented by the majority of sequences (i.e. roughly one third of all sequences). The distribution and sequence representation for *gtf* was different (Figure 2). Here, only 16 OTUs (52%) were unique to one country and they were represented by only 67 sequences (9% of all sequences). Six OTUs (19%) were shared between any two countries, accounting for 11% of all sequences. 22% of OTUs were present in three, four or five countries and accounted together for 26% of all sequences. There was one *gtf*-OTU present in 6 countries and one *gtf*-OTU present in all seven countries. Both of these OTUs were represented by roughly equal numbers of sequences which together made up 53% of all sequences in the *gtf*-dataset (Figure 2).

The sources of genetic variation as calculated via AMOVA were quite distinctive among the three genes (Table 3). Most variation in *gdh* and ITS were due to sequence diversity within individuals, followed by sequence variations among individuals with virtually little sequence variation due to geographic origin. Conversely, sequence variation among individuals made up the highest source of variation in the case of *gtf*. Most importantly, geographic origin accounted for 10.7% of the variance.

**Table 3:** AMOVA values calculated at three hierarchical levels for seven countries

**Gene**	***gdh***		***gtf***		**ITS**	
	**A**	**B**	**A**	**B**	**A**	**B**
Among countries	0.71	0.72	8.76	10.72	n.d.	−0.09
Among individuals within countries	21.98	22.27	54.51	57.52	n.d.	19.41
Within individuals	77.32	77.00	36.73	31.75	n.d.	80.67
*P*^*1*^	0	0	0	0	n.d.	0.5

A: Calculation based on unique sequences.

B: Calculation based on OTUs defined at >99% level.

n.d.: not determined.

^1^P-values were determined based on permutation analysis using 1,000 permutations.

As the *gdh*- and ITS-dataset revealed no geographic structure further analysis focused on the *gtf*, for which five additional locations (i.e. AR, BO, CA, LO, and SO, again 10 individuals each) were completed and jointly analyzed with the former data, resulting in the full data set comprising 12 world-wide regions. This dataset comprised 1,168 *gtf*-sequences, including 332 unique sequences which could be further classified into 57 distinct OTUs (defined at >99% identity). Recalculation of the AMOVA based on these OTUs revealed a partitioning of genetic variation into 34.5% within individuals, 57.3% among individuals per country and 8.2% among countries.

**Figure 3:**
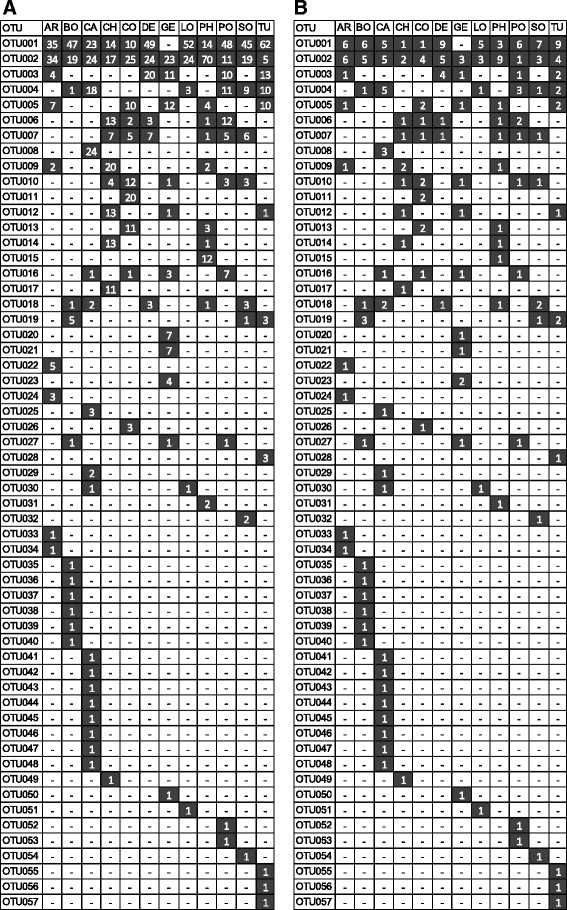
**Abundance distribution of*gtf*-OTUs across all 12 geographic locations, A) # of sequences, B) # of individuals positive for that OTU.** AR = Argentina, BO = Bolivia, CA = California, CH = China, CO = Congo, DE = Germany, GE = Georgia, LO = Louisiana, PH = Philippines, PO = Poland, SO = South Africa, TU = Turkey.

The global distribution pattern revealed two OTUs (OTU001 and OTU002) as conspicuously dominating in prevalence and abundance, whereas the remaining 55 OTUs were notably less abundant and either scattered across two to six countries or found only in a single country (Figure 3). OTU001 was present in all but one country and accounted for 34% of sequences (Figure 3A) with highest prevalence in DE (9 individuals) and lowest in CH and CO (one individual each), Figure 3B. Likewise, OTU002 was ubiquitously present and accounted for 25% of sequences. Highest prevalence was seen in PH (9 individuals) and lowest in PO (1 individual). The abundance of all other OTUs strongly decreased and so did their prevalence across and within countries. For instance, the abundance of OTU003 and OUT004 accounted for only 5.0% and 4.5% of all sequences, yet they were distributed over 5 and 6 countries, respectively. 36 OTUs were found only in a single country, most of which were represented with very few clone sequences. Exceptions to this were OTU008 (CA), OTU011 (CO), OTU015 (PH), and OTU017 (CH), confined to a single country but represented by a relative large number of clone sequences (i.e. 11 to 24).

**Figure 4:**
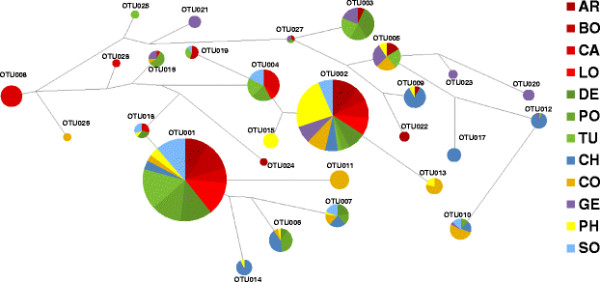
**Network analysis based of 28*gtf*-OTUs obtained from 12 geographic locations.** AR = Argentina, BO = Bolivia, CA = California, CH = China, CO = Congo, DE = Germany, GE = Georgia, LO = Louisiana, PH = Philippines, PO = Poland, SO = South Africa, TU = Turkey.

Haplotype Network analysis based on those 28 OTUs, represented by at least three clone sequences, revealed some plausible associations in that OTUs shared by multiple countries were generally linked with OTUs shared by largely the same set of countries, e.g. the pairs OTU001/OTU018, OTU002/OTU004, OTU003/OTU027, OTU004/OTU019 (Figure 4). The two major OTUs (OTU001 and OTU002) both of which were dominated by the American and European countries, were not directly connected to each other but instead were linked to a number of minor OTUs. Most of these minor OTUs lacked sequences from American individuals (e.g. OTU006, 007, 011, 013, and 014). Except for OTU011, all OTUs unique to a given country were only connected via one or two nodes with other OTUs.

**Figure 5:**
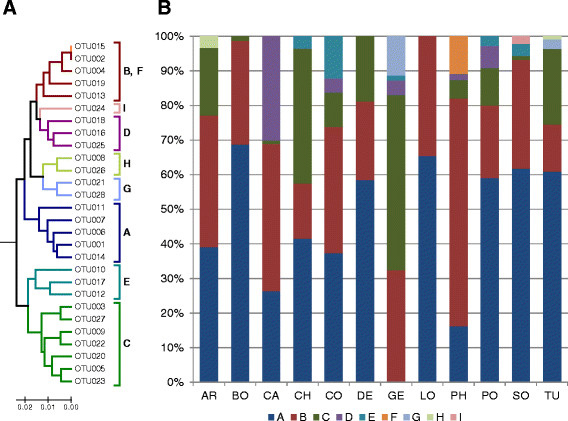
***Gtf*-subclusters. (A)** Phylogenetic UPGMA tree of predominant *gtf*-variants defined as OTUs at 99% identitiy level with the sub-clusters A to I defined at 97% identity level. Note, that OTU015 (sub-cluster F) differs from OTU002 by a deletion of 54-nucleotides, which is not visible in the treeing analysis. For further information, see main text. The scale bar represents 2% nucleotide differences; **(B)** Relative abundance of *gtf*-sub-clusters (A to I) across all 12 geographic locations. AR = Argentina, BO = Bolivia, CA = California, CH = China, CO = Congo, DE = Germany, GE = Georgia, LO = Louisiana, PH = Philippines, PO = Poland, SO = South Africa, TU = Turkey.

Phylogenetic tree reconstruction of those 28 OTUs, represented by at least three clone sequences revealed two major branches, which could be subdivided into 9 sub-clusters (A to I, defined at a >97% identity level; Figure 5A). The two most abundant OTUs (OTU001 and OTU002) group separately (i.e. in sub-clusters A and B), and were each affiliated with an additional four OTUs. While BO and LO were almost entirely composed of sub-cluster A and B, these two sub-clusters made up at least 55% of all other countries (except for GE which was devoid of sub-cluster A; Figure 5B). The remaining sub-clusters varied in abundance among countries. For instance, CH and GE were characterized by a high abundance of sub-cluster C, which in turn was very low in BO, CA, SO, and completely absent in LO. Likewise, sub-cluster D was strongly dominant in CA, moderately present in CO, GE, PH, and PO but otherwise absent in all other countries. Sub-cluster E showed the highest abundance in CO and was otherwise confined in low abundance to CH, GE, PO and SO (Figure 5B). Sub-cluster F, composed of a single OTU (i.e. OTU0015, which was identical to OTU002, but with a 54-nucleotide deletion that maintained the reading frame) was exclusively identified in one individual from PH. In order to rule out a possible PCR-artifact, this sample was re-amplified by PCR and new clones analyzed. Again, the identical sequence with a 54-nucleotide deletion was obtained in the new clone library, confirming the authenticity of this sequence variant. Authenticity of this 54-nucleotide deletion was further supported by the fact that it was observed in another (rare) sequence variant in an individual from SO (not displayed in Figure 5A). Sub-cluster G was only found in GE and TU, sub-cluster H only in AR and TU, and sub-cluster I only in SO (Figure 5B).

**Figure 6:**
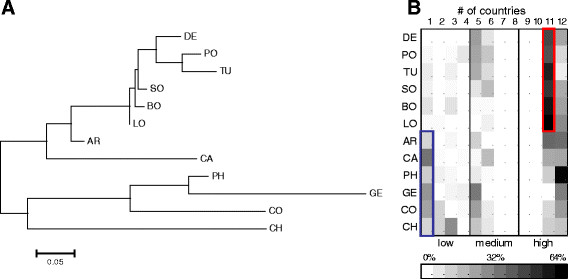
***GTF*-OTU-distribution. (A)** Dendrogram displaying the assignment of 12 geographic regions using the Pearson Distance correlation analysis based on *gtf*-OTU-distribution; **(B)** heatplot displaying OTU-types and their distribution across 12 geographic locations. OTU-types are defined according to their presence in only one or multiple countries (white, 0% presence, black, 64% presence). By way of example, the upper six countries are characterized by dominance of OTUs, which are distributed over 11 countries (red square), while the lower six countries are comparably high in OTUs confined to a single geographic location (blue square). AR = Argentina, BO = Bolivia, CA = California, CH = China, CO = Congo, DE = Germany, GE = Georgia, LO = Louisiana, PH = Philippines, PO = Poland, SO = South Africa, TU = Turkey.

We next mapped the 12 countries based on the distribution pattern of all 57 *gtf*-OTUs using the Pearson Distance analysis. Two major branches emerged, one displaying the European and American countries along with SO, the other displaying the Asian countries along with GE and CO (Figure 6A). While AR and CA were characterized by distinct lineages within the former branch, the countries BO, LO and SO grouped close together next to the European cluster. In contrast, the Asian countries and CO in the second branch were all separated by very long branches. The overall topology of this cluster tree correlated with the OTU-types displayed in Figure 6B. Countries in the first branch were less characterized by rare OTUs but instead were strongly dominated by OTUs present in at least 11 countries (red square, Figure 6B), except for AR and CA (which explains their somewhat unique positions). The pattern for the countries from the second branch was different in that the ratio of rare and common OTUs was more balanced, with a comparably high number of OTUs confined to a single country (blue square, Figure 6B). Two further patterns emerge in Figure 6B: first, the countries from the American continent (AR, BO, CA, and LO) are comparably depauperate in OTUs, which are distributed over four to six countries; second, the entire *gtf*-gene dataset was devoid of any OTU distributed across seven, eight, nine or ten countries (Figure 6B).

**Figure 7:**
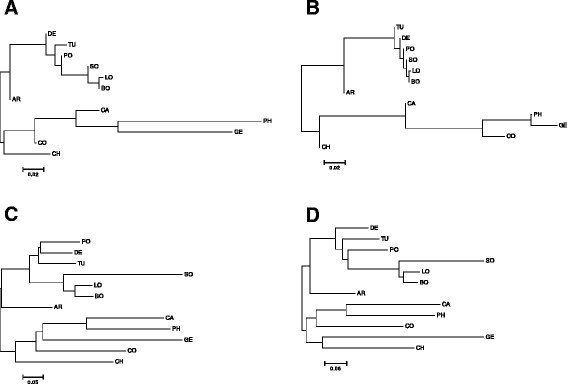
**(Upper panel) Neighbor-joining trees based on pairwise Fst-Values using DNA (A) or protein (B) sequence; (Lower panel) Neighbor-joining trees based on UniFrac analysis with DNA (C) or protein (D) sequence.** AR = Argentina, BO = Bolivia, CA = California, CH = China, CO = Congo, DE = Germany, GE = Georgia, LO = Louisiana, PH = Philippines, PO = Poland, SO = South Africa, TU = Turkey.

Countries were also mapped based on pair-wise Fst-values calculated from nucleic acid and deduced amino acid sequences (Figure 7A and B). Both cluster trees largely resembled each other and mirrored the tree that was obtained with the Pearson distance calculation, except for CA, which grouped together with the Asian/CO-branch when Fst-values were used. The distances among countries were markedly lower in the protein-based tree for the countries BO, DE, LO, PO, SO, TU, and CO, GE, and PH, respectively (Figure 7B).

The tree topology based on pair-wise Fst-values was largely confirmed with UniFrac-based clustering of countries calculated based on phylogenetic trees using nucleic-acid and deduced amino-acid sequences (Figure 7C and D). However, in the UniFrac analysis the countries were generally separated by longer branches.

The overall influence of the OTU definition at >99% identity level for the assignment of geographic locations was verified by re-defining OTUs using a 97% and 98% threshold, respectively. The resulting cluster-trees based on pair-wise Fst-values were highly similar to each other and to the trees shown in Figure 7, except for the position of CH which now grouped in the upper branch (Additional file 2: Figure S2).

**Figure 8:**
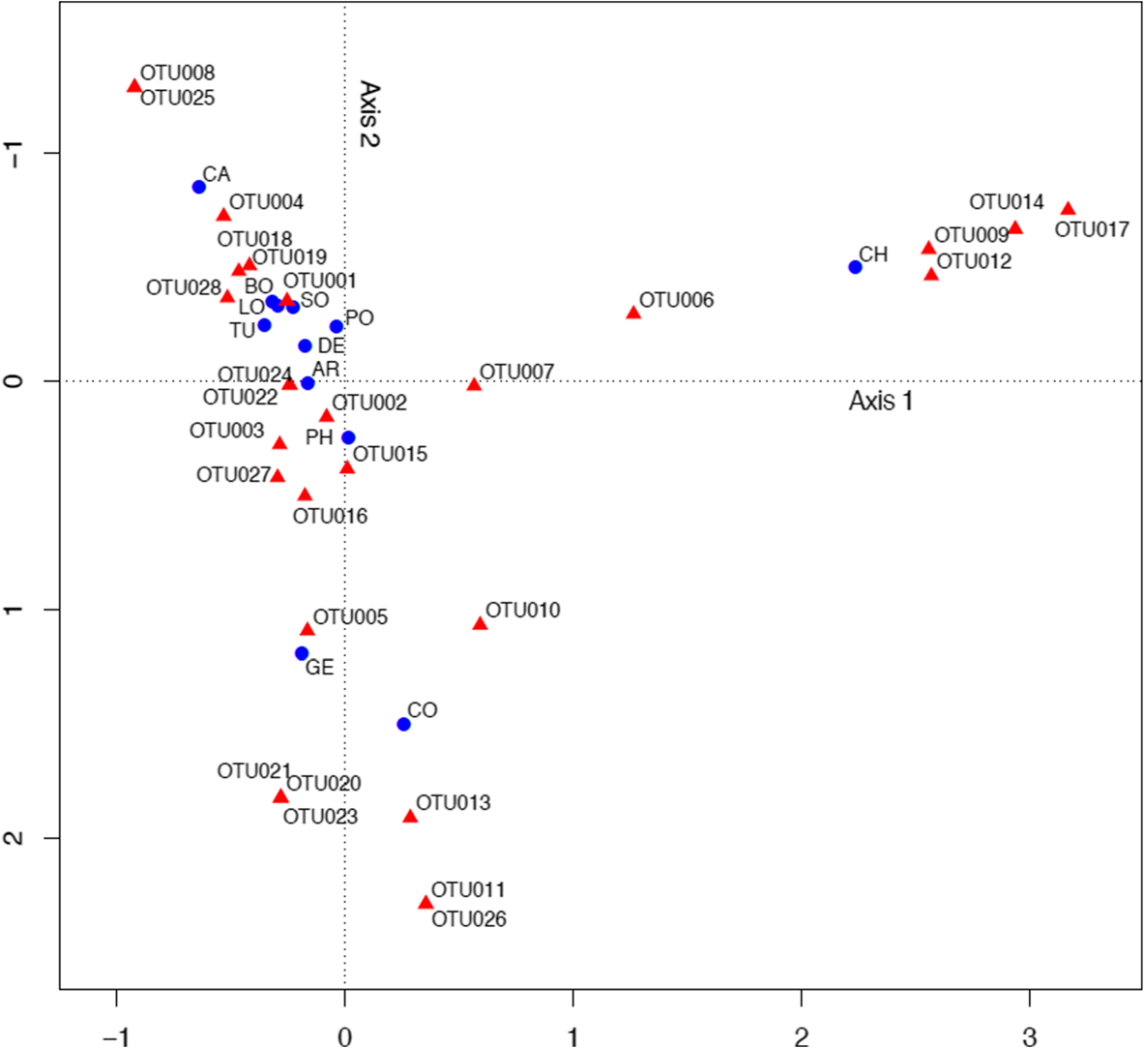
**Relationship between OTUs and countries calculated with correspondence analysis, based on the data presented in Figure**3**.** AR = Argentina, BO = Bolivia, CA = California, CH = China, CO = Congo, DE = Germany, GE = Georgia, LO = Louisiana, PH = Philippines, PO = Poland, SO = South Africa, TU = Turkey.

Correspondence analysis confirmed the tight clustering of the European and American countries, with a moderate proximity of PH to those countries (Figure 8). CH is a clear outlier due to several OTUs, as are CO and GE.

**Figure 9:**
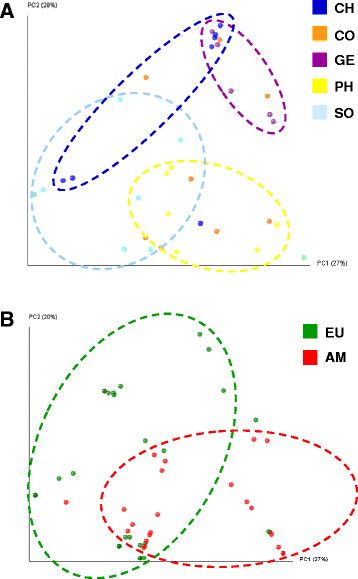
**Two-dimensional principle coordinate analysis based on UniFrac distances.** Each colored dot is an individual of the following countries (A) CH, CO, GE, PH, and SO; (B) EU = European countries DE, PO, and TU; AM = American countries/states AR, BO, CA, LO; Dashed circles indicate the tentative grouping of individuals from the same geographic location; AR = Argentina, BO = Bolivia, CA = California, CH = China, CO = Congo, DE = Germany, GE = Georgia, LO = Louisiana, PH = Philippines, PO = Poland, SO = South Africa, TU = Turkey.

We also used UniFrac-distances calculated from phylogenetic trees for displaying the relatedness of individuals based on the variation of the *gtf*. As can be seen in Figure 9A individuals from any given country did not exclusively group together, however certain tendencies were apparent. For instance, individuals from PH grouped comparably close together and were particularly distinct from GE and most individuals of CH. Individuals from GE grouped also tight together and showed some overlap with CH but no overlap with SO. In this plot the most scattered individuals were those from CO. Individuals from America and Europe were also largely scattered and therefore omitted in Figure 9A for transparency. However, re-assignment of corresponding countries at the continent level showed some separation between individuals from Europe and America (Figure 9B).

## Discussion

### General considerations

Analyses of the human microbiome have strongly progressed in recent years. Cultivation-independent approaches (particularly metagenomics) have provided insights into the tremendous diversity of microbial species, many of which were previously unknown [47],[48] From a medical perspective it seems indisputable that the type of bacterial species, their composition and interactions affect in many (yet to be elucidated) ways our health or predisposition for various diseases [49],[50]. However, a major hurdle towards an advanced comprehension of the human microbiome is its huge intra- and interpersonal variation as a reflection of age, gender, immune system, physiologic status, diet and hygiene, along with many other cultural or lifestyle factors. This complexity has led to an emerging view for the need for interdisciplinary research linking human microbial ecology with various subfields of anthropology [51],[52]. For instance, comparably little is known so far about microbiome variation among individuals from different geographic or ethnic origins. If parts of the microbiome are structured due to geography or ethnicity this lends itself to a more “applied” research question, namely: can we deduce human population relationships and migration history based on the genetic variations within their microbiomes? This is a worthwhile question given that there is still controversy over how humans have migrated in prehistoric times [53],[54], and given that studies on the genetic architecture of human microbes (in particular on *H. pylori*) have helped resolve some of these issues [4],[5],[10]. With the consideration of a body habitat more conveniently accessible than the stomach, Nasidze et al. [25] recently provided a comprehensive 16S rRNA gene based survey of the salivary microbiota by comparing healthy individuals from 12 geographic regions world wide. A major conclusion of this study was that variations among individuals are persistently/consistently high independently from the geographic distance of any two compared individuals. Hence, no geographic structure of the salivary microbiome at the genus and species level could be ascertained.

However, the fact that many bacterial species are generally represented by numerous clonal variants indistinguishable by the 16S rRNA gene prompted us to re-investigate the above samples with a focus on protein-coding genes and the ITS-region, which are less conserved than the 16S rRNA gene. Our aim was to find out whether or not clonal variants of oral bacterial species correlate with the geographic region/country of their hosts, and we selected the above samples, because of the apparent lack of a geographic structure at the 16S rRNA gene level. In addition, these samples suited our purpose as they were collected independently from general health or dental status and lifestyle of the individuals, such as gender, age, diet or hygiene etc. We believe this to be important, because if a bacterial gene is applicable at all for inferring human population relationships its geographic signature (if existing) must be robust enough to overcome at least partially individual-specific traits or behavior. Only such a bacterial gene would make large-scale population analyses with thousands of individuals feasible, because with increasing number of individuals (involving family members, such as grandparents, parents and children) the consistent controlling for the above factors becomes more and more difficult if not entirely impossible. While individuals of the currently tested sample set were characterized by multiple clone variants, we did not observe a correlation with geography for the genes *gdh* and ITS. Conversely, the gene *gtf* exhibited a geographic signature of around 8% for the 12 countries analyzed, which is a notable value because it compares to the human genome structure from different continents [55]. Hence of the three genes tested one exhibited a promising robustness over individual-specific factors, such as diet and hygiene. However, it should be noted that if we had controlled for these individual-specific factors, we may have found significant geographic signature values for the other two genes analyzed.

The multiple clones of bacterial species that can be found in one individual may reflect neutral variants or may be the result of adaptation to the numerous ecological niches in the upper respiratory tract. However, at a given time point and at one particular oral site only few or even only one clonal type might dominate, while others are below the level detectable by traditional culture techniques [2]. Consequently, saliva, which does not contain its own microflora but rather constitutes a representation of other oral sites [13], may also harbor a limited number of highly abundant clonal variants. With restriction to ten clones per individual we aimed at those predominating sequence types rather than assessing the entire clonal diversity. This is because with the long-term objective of large-scale population studies it would be much more practical to retrieve meaningful information with abundant gene variants rather than having to perform an in-depth sequencing for every individual. In addition, if we consider that intra-individual clonal types are more closely related to each other than those found between individuals, the dominating sequence type per individual should be a fair representative for distinguishing clones obtained from geographically different regions or different ethnic groups. In any case, our approach is still more sensitive than a cultivation-based approach, since most clonal types escape cultivation efforts anyway due to their low abundances [2].

### Comparison of *gdh*, *gtf*, and ITS

Lack of a geographic structure in the *gdh* and ITS genes contrasts the fact that more polymorphic sites and ultimately much more sequence variants were available for these genes compared to *gtf*. For instance, the number of *gdh*- and ITS-OTUs per country exceeded the number of *gtf*-OTUs by a factor of four to five (Table 2), which may then result in a higher amount of information for proper distinction of geographic locations. This assumption is supported by Figure 2, where both *gdh* and ITS suggest that each country is unique owing to the large proportions of OTUs only present in a single country. Nonetheless, despite the higher number of country-specific OTUs, when looking at polymorphic sites the AMOVA revealed that most of the genetic variation of *gdh* and ITS is largely explained by the high within-individual variation (Table 3), which is also supported by the higher richness in OTUs per individual (Figure 1). Conversely, the proportion of shared OTUs among two or more countries is strikingly higher for *gtf* (Figure 2), and consequently, it would be fair to assume that the *gtf* gene tentatively reflects similarities rather than differences among the countries (resulting in a low geographic signal). However, despite the relative high proportion of common OTUs the precise combination (distribution pattern) of shared OTUs (Figure 3) along with a comparably low within-individual genetic variation (Table 3) has apparently led to the preservation of some degree of geographic structure of the *gtf* gene during its diversification and adaptation to the host.

An explanation for this could be that the glucosyltransferases (GTFs) play critical and complex roles in the development of dental plaque. For instance, GTFs of cariogenic *S. mutans* not only adsorb to enamel synthesizing glucans but also adsorb to surfaces of other oral microorganisms that do not encode for *gtf-*genes (e.g. *Actinomyces* spp.), thereby converting them to glucan producers [56],[57]. Although mainly described for *S. mutans* it is plausible to assume that the GTFs of other oral streptococci are engaged in similar ecological interactions with the oral microbiota. This means, that the adsorption of GTFs to a multitude of oral bacterial species may only work with the existence of universal binding sites, which has to come along with some degree of conservation of the *gtf*-gene for proper biofilm formation in the human oral cavity. Recently reported evidence of negative selection acting upon the *gtf* genes of *S. mutans* supports this assumption [34].

Another possible explanation for the differential characteristics of the three gene datasets could be that with each gene a different spectrum of species was covered. That is, all *gtf*-variants analyzed in this study could be assigned to a single species, namely *S. oralis*, while the *gdh*-variants covered three species of the mitis-streptococci-group (i.e. *S. oralis*, *S. mitis*, and *S. infantis*). In addition, with ITS we covered *F. nucleatum* and its close relative *F. periodonticum*. If closely related bacterial species co-evolved differently with the host their joint analysis would probably blur a geographic signature. However, we think that no such scenario has affected our data interpretation, as we performed the analysis also with a separated, species-specific dataset. To this end publicly available reference sequences from bacterial culture isolates were used for joint phylogenetic tree analysis with our sequence data. This enabled an unambiguous assignment of the *gdh*-OTUs and ITS-OTUs to a given species (data not shown) and those “Species-specific” OTUs could then be analyzed as single datasets. Based on AMOVA the geographic signature (that is, the “among countries genetic variation”) remained close to zero for the split datasets of *gdh* and ITS. Although the overall statistical power was reduced in this case (due to the lower number of sequences analyzed per species) we believe that a possible geographic signature of the *gdh* gene and the ITS may only be revealed when looking at the entire clonal diversity per individual. This, however, would be rather impractical for the reasons stated above. Furthermore, since the *gtf* gene already “scored best” based on the abundant clonal types, analyzing the full breadth of *gtf* gene diversity appears to be more promising and work is currently underway characterizing the *gtf* gene by massive in-depth parallel sequencing.

### The geographic signature of *gtf*

Interestingly, the two most abundant clonal variants of *gtf* (OTU001 and OTU002) are distributed world-wide, yet in no country with a prevalence of 100 percent (Figure 3). In fact, the prevalence fluctuated from one up to nine positive individuals depending on country. Clearly, these clonal variants may be in fact present in every single individual, but were simply by chance not picked up in the clones that were sequenced. However, lack of detection in all samples from GE, nine individuals each from CH and CO (in the case of OTU001) and in nine individuals from PO (in the case of OTU002) suggests that these clonal variants (even if present) are at least markedly less abundant in individuals from the corresponding countries. This implies that they likely might have been overlooked also by traditional culture techniques in those individuals. Hence, while apparently successfully adapted to the human host as reflected by the world-wide ubiquity, some individual factors may preclude the clonal variants OTU001 and OTU002 to colonize every single person in high abundance. Successful colonization of the human host appears to drop dramatically for all subsequent clonal variants (i.e. OTU003-OTU057, Figure 3). These OTUs are scattered among few countries and only with few individuals positive therein. This indicates on the one hand that these clonal variants are rare in prevalence and on the other hand it indicates that more individuals need to be analyzed in order to see which OTUs are truly unique or shared among countries. However, the haplotype network shows that many shared OTUs are connected with other OTUs also shared by the same or a similar range of countries (Figure 4). Such a distribution pattern would probably not emerge, if the geographic regions were massively undersampled. Interestingly, the haplotype network also mirrors some plausible geographical arrangements in that OTUs predominantly shared among individuals from CH or American individuals tentatively are located at opposite sites of the net (e.g. OTU009, OTU010, OTU012, and OTU017 versus OTU004, OTU008, OTU018, and OTU28).

Even when just looking at OTU distribution (that is without sequence information) the Pearson correlation analysis displayed some plausible/fundamental relationships among countries (Figure 6). European countries form one tight cluster, and group with those historically influenced by Europe (i.e. America and SO) and this branch is clearly separated from CO, GE and Asian countries. Figure 6B clearly explains why, namely the first branch is much more strongly characterized by the pre-dominance of the two ubiquitous OTUs (OTU001 and OTU002, with a prevalence in 11 and 12 countries, respectively) while the second branch reflects an elevated number of rare OTUs. Those relationships among countries based on the distribution pattern of OTUs are also largely confirmed based on precise sequence information using pair-wise Fst-values or UniFrac based interpretations of phylogenetic trees, albeit with some minor alterations in the tree topologies (Figure 7). In particular, UniFrac analysis indicates that underlying phylogenetic trees are not heavily blurred by homologous recombination events which most likely occurred at a local rather than at a global scale, thereby probably even contributing to an increased geographic signature. The fact that the topology of the countries in the cluster-trees remained stable even when OTUs were defined at an identity level of 97% (i.e. subclusters A to I, Figure 5, and Additional file 2: Figure S2) indicates the overall robustness of the sequence data set. Even at this relatively high threshold level, the proportions of the resulting nine subclusters (A to I) are quite distinctive among countries with some variants restricted to one or few countries (Figure 5). The relative low abundance of the country-specific subclusters however again indicates that more than ten individuals per country are needed for a proper comparison and differentiation of countries. Another interesting insight stems from the correspondence analysis (Figure 8), which shows, that CH, CO, and GE are distinct from the European/American countries based on several OTUs. These results are consistent with the OTU abundances (Figure 3). Moreover, the OTUs that characterize these outlying countries are not closely-related to one another (Figure 4, Figure 5), indicating that each outlier is characterized by a set of disparate OTUs, rather than by clonal expansion of closely-related OTUs.

In the current study, we did not test the sequence data for potential chimeric artifacts formed during PCR, because such artifacts are indistinguishable from true sequence variants as a result of homologous recombination. However, it is plausible to assume that such PCR artifacts (if they exist) lead to OTUs that are at best abundant in one particular sample or individual, but not spread as identical OTUs among individuals. This means that those OTUs that occur only in one individual, as displayed in Figure 3, are, if any, the most likely candidates to represent chimeric molecules. Therefore, we performed AMOVA and calculated a cluster-tree based on pair-wise Fst-values without those putative chimeric OTUs. The proportion of the total variance due to differences between geographic regions remained at about 8%, and the Fst-cluster tree was highly identical to those shown in Figure 7 (Additional file 3: Figure S3). Hence, our overall interpretation of the data is not distorted by the possible presence of chimeric molecules. Instead, such artifacts would be expected to increase within-individual genetic diversity, thereby reducing the geographic signal. In addition, the haplotype network (Figure 4) has no reticulations, indicating that there is no detectable recombination in the dataset.

In order to relate our work with data from *H. pylori* we compared the pairwise Fst-values obtained for the 12 countries (Additional file 4: Table S1) with those previously described for human populations from countries across Europe, Middle East and Asia [58], based on *H. pylori.* The Fst-values in our study range from 0.002 to 0.38 (median 0.11, n = 66) whereas in the case of *H. pylori* such values only range from 0.002 to 0.13 (median 0.04, n = 210) [58]. Although mainly different countries were analyzed for *H. pylori*, the notably higher range of Fst-values observed in our study can be viewed as a reasonable estimation of the overall informational potential of the *gtf* gene*.* In addition, three countries in the *H. pylori* study above coincide with our study, namely Germany, Turkey and the Philippines allowing a direct comparison. And in fact, Fst values based on *gtf* are about four times higher than those based on *H. pylori* for the pairs (DE-PH and PH-TU) but, interestingly, two times lower for the pair DE-TU (Table 4). Although more data are needed for valid conclusions the particularly high values observed in conjunction with PH indicates quite distinctive oral bacteria in the individuals from the Philippines.

**Table 4:** **Pairwise Fst-values based on the** ***gtf*** **gene (in brackets) and** ***H. pylori***^**1**^

	**DE**	**PH**	**TU**
DE	-	-	-
PH	0.062 (0.304)	-	-
TU	0.022 (0.009)	0.106 (0.383)	*-*

^1^Data from *H. pylori* where taken from [58].

DE = Germany, PH = Philippines, TU = Turkey.

## Conclusions

We have shown here that protein-coding bacterial genes from human saliva (such as the *gtf* gene from *S. oralis*) exhibit a geographic signature, which could potentially be used for addressing previously unresolved human migration events. Clearly, in the current study only a relatively short fragment (around 330 bp) of the *gtf* gene was analyzed, which means that a sequence dataset solely based thereon hardly provides sufficient information for proper distinction of human populations. This may, in part explain, why individuals from a country did only group together tentatively (Figure 9). A higher resolution with oral bacterial genes would be achieved through the accumulation of sequence information as it is done with *H. pylori*, for which usually about 4,500 bp, (seven housekeeping genes and two virulence genes) are routinely analyzed [10]. More information could potentially be obtained through joint analysis of other *gtf* genes and homologous genes from multiple streptococci. Even non-related genes from other oral bacteria (with a geographic signature) could be used in concert, as long as the mere OTU-distribution pattern (e.g. analyzed with the Pearson correlation) allows meaningful inferences. In other words, once tested successfully as single genes, a set of several oral bacterial genes (related or even non-related) could be assembled and the distribution pattern jointly compared among human individuals. This would be in rough analogy to the concatenation of different genes from microbial isolates in order to compare them via MLST. Owing to the functional richness of the oral microbiome [59] a sufficiently high number of biogeographically relevant genes may eventually be identified for the above purpose. The current study provides proof-of-principle and points towards investigations to establish specific saliva bacteria and their genes as a useful alternative to *H. pylori* for investigating human population structure and migrations.

## Competing interests

The authors declare that they have no competing interests.

## Authors’ contributions

HPH, MS and GC designed the study. AS, HS, and OK carried out the laboratory work. KH, JL, HPH, and MS analyzed the data. HPH, KH, GC and MS wrote the manuscript. All authors read and approved the manuscript.

## Additional files

## Supplementary Material

Additional file 1: Figure S1. Map of the 12 sampling regions. AR = Argentina, BO = Bolivia, CA = California, CH = China, CO = Congo, DE = Germany, GE = Georgia, LO = Louisiana, PH = Philippines, PO = Poland, SO = South Africa, TU = Turkey. Click here for file

Additional file 2: Figure S2. Neighbor-joining trees based on Pairwise-Fst-values of the gtf gene variants defined as OTUs at 98% identity level (A) and at 97% identitiy level (B). AR = Argentina, BO = Bolivia, CA = California, CH = China, CO = Congo, DE = Germany, GE = Georgia, LO = Louisiana, PH = Philippines, PO = Poland, SO = South Africa, TU = Turkey. Click here for file

Additional file 3: Figure S3. Neighbor-joining trees based on Pairwise-Fst-values of the gtf gene variants defined as OTUs at 99% identity level, under omission of all OTUs that were unique to a single individual. This calculation was performed to test the impact of putative chimeras on the tree topology. AR = Argentina, BO = Bolivia, CA = California, CH = China, CO = Congo, DE = Germany, GE = Georgia, LO = Louisiana, PH = Philippines, PO = Poland, SO = South Africa, TU = Turkey. Click here for file

Additional file 4: Table S1. Pairwise Fst-values based on the gtf gene for the 12 analyzed geographic regions. Click here for file
