# Supplementary material for: Global analysis of saliva as a source of bacterial genes for insights into human population structure and migration studies
Source: BMC Evol Biol. 2014 Aug 22;14:190. doi: 10.1186/s12862-014-0190-3 (PMC4360258; doi:10.1186/s12862-014-0190-3)
Supplement: Additional file 1: Figure S1. — Map of the 12 sampling regions. AR = Argentina, BO = Bolivia, CA = California, CH = China, CO = Congo, DE = Germany, GE = Georgia, LO = Louisiana, PH = Philippines, PO = Poland, SO = South Africa, TU = Turkey. [file s12862-014-0190-3-S1.pdf]

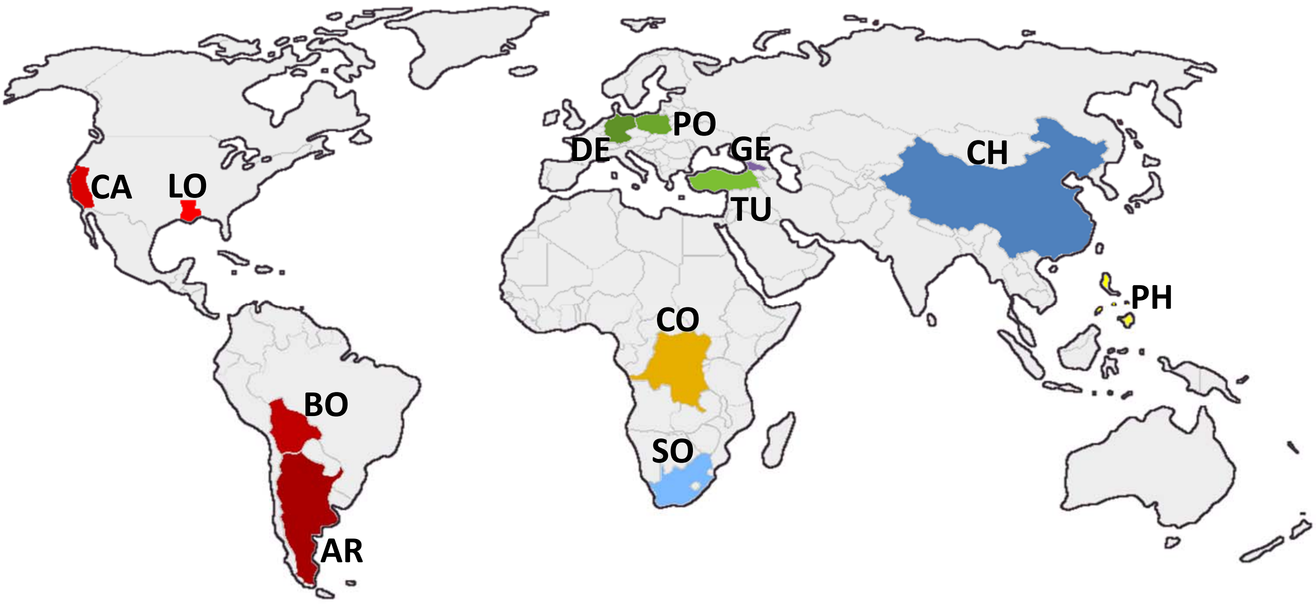

Fig. S1: Map of the 12 sampling regions. AR = Argentina, BO = Bolivia, CA = California, CH = China, CO = Congo, DE = Germany, GE = Georgia, LO = Louisiana, PH = Philippines, PO = Poland, SO = South Africa, TU = Turkey
