# Supplementary material for: Global analysis of saliva as a source of bacterial genes for insights into human population structure and migration studies
Source: BMC Evol Biol. 2014 Aug 22;14:190. doi: 10.1186/s12862-014-0190-3 (PMC4360258; doi:10.1186/s12862-014-0190-3)
Supplement: Additional file 3: Figure S3. — Neighbor-joining trees based on Pairwise-Fst-values of the gtf gene variants defined as OTUs at 99% identity level, under omission of all OTUs that were unique to a single individual. This calculation was performed to test the impact of putative chimeras on the tree topology. AR = Argentina, BO = Bolivia, CA = California, CH = China, CO = Congo, DE = Germany, GE = Georgia, LO = Louisiana, PH = Philippines, PO = Poland, SO = South Africa, TU = Turkey. [file s12862-014-0190-3-S3.pdf]

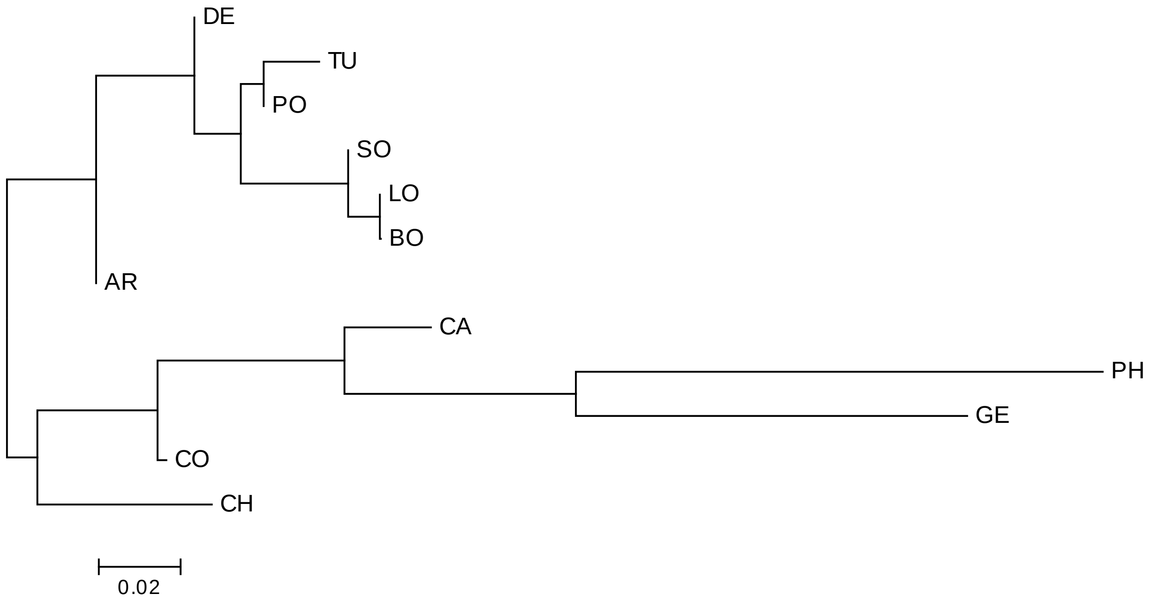

Fig. S3: Neighbor-joining trees based on Pairwise-Fst-values of the gtf gene variants defined as OTUs at 99% identity level, under omission of all OTUs that were unique to a single individual. This calculation was performed to test the impact of putative chimers on the tree topology.
AR = Argentina, BO = Bolivia, CA = California, CH = China, CO = Congo, DE = Germany, GE = Georgia, LO = Louisiana, PH = Philippines, PO = Poland, SO = South Africa, TU = Turkey
