# Supplementary material for: Global analysis of saliva as a source of bacterial genes for insights into human population structure and migration studies
Source: BMC Evol Biol. 2014 Aug 22;14:190. doi: 10.1186/s12862-014-0190-3 (PMC4360258; doi:10.1186/s12862-014-0190-3)
Supplement: Additional file 4: Table S1. — Pairwise Fst-values based on the gtf gene for the 12 analyzed geographic regions. [file s12862-014-0190-3-S4.pdf]

**Table S1:** Pairwise Fst-values based on the *gtf* gene for the 12 analyzed geographic regions

| | AR | BO | CA | CH | CO | DE | GE | LO | PH | PO | SO | TU |
|---|---|---|---|---|---|---|---|---|---|---|---|---|
| AR | 0,0000 | | | | | | | | | | | |
| BO | 0,0560 | 0,0000 | | | | | | | | | | |
| CA | 0,0777 | 0,1555 | 0,0000 | | | | | | | | | |
| CH | 0,0632 | 0,1472 | 0,1905 | 0,0000 | | | | | | | | |
| CO | 0,0427 | 0,1138 | 0,0957 | 0,0731 | 0,0000 | | | | | | | |
| DE | 0,0282 | 0,0435 | 0,1339 | 0,0724 | 0,0821 | 0,0000 | | | | | | |
| GE | 0,1779 | 0,3641 | 0,2067 | 0,1724 | 0,1630 | 0,2374 | 0,0000 | | | | | |
| LO | 0,0572 | -0,0101 | 0,1577 | 0,1565 | 0,1205 | 0,0509 | 0,3725 | 0,0000 | | | | |
| PH | 0,1885 | 0,3525 | 0,1668 | 0,2801 | 0,1822 | 0,3040 | 0,2481 | 0,3502 | 0,0000 | | | |
| PO | 0,0516 | 0,0277 | 0,1452 | 0,0893 | 0,0904 | 0,0115 | 0,2923 | 0,0378 | 0,3442 | 0,0000 | | |
| SO | 0,0415 | -0,0030 | 0,1363 | 0,1165 | 0,0858 | 0,0307 | 0,3244 | 0,0019 | 0,3260 | 0,0118 | 0,0000 | |
| TU | 0,0593 | 0,0524 | 0,1752 | 0,0928 | 0,1088 | 0,0088 | 0,2872 | 0,0653 | 0,3830 | 0,0092 | 0,0364 | 0,0000 |
